# The Professional Phagocyte *Dictyostelium discoideum* as a Model Host for Bacterial Pathogens

**DOI:** 10.2174/138945011795677782

**Published:** 2011-06

**Authors:** Salvatore Bozzaro, Ludwig Eichinger

**Affiliations:** 1 Department of Clinical and Biological Sciences, University of Turin, Ospedale S. Luigi, 10043 Orbassano, Italy; 2 Centre for Biochemistry, Medical Faculty, University of Cologne, 50931 Cologne, Germany

**Keywords:** Amoeba, *Dictyostelium discoideum*, drug targets, functional analysis, infection, *Legionella pneumophila*, model organism, *Mycobacteria*, pathogen, *Salmonella typhimurium*, social amoeba, virulence factor.

## Abstract

The use of simple hosts such as *Dictyostelium discoideum* in the study of host pathogen interactions offers a number of advantages and has steadily increased in recent years. Infection-specific genes can often only be studied in a very limited way in man and even in the mouse model their analysis is usually expensive, time consuming and technically challenging or sometimes even impossible. In contrast, their functional analysis in *D. discoideum* and other simple model organisms is often easier, faster and cheaper. Because host-pathogen interactions necessarily involve two organisms, it is desirable to be able to genetically manipulate both the pathogen and its host. Particularly suited are those hosts, like *D. discoideum*, whose genome sequence is known and annotated and for which excellent genetic and cell biological tools are available in order to dissect the complex crosstalk between host and pathogen. The review focusses on host-pathogen interactions of *D. discoideum* with *Legionella pneumophila, mycobacteria*, and *Salmonella typhimurium* which replicate intracellularly.

## INTRODUCTION

*D. discoideum* is a fascinating member of the amoebozoa, whose natural habitat is deciduous forest soil and decaying leaves, where the amoebae feed on bacteria and yeast and grow as separate, independent, single cells. Upon depletion of food, the cells undergo aggregation and cell differentiation, giving rise to a multi-cellular organism made up of different cell types [1]. The organism offers unique advantages for studying fundamental cellular processes with powerful molecular genetic, biochemical, and cell biological tools [2]. These processes include cell motility, chemotaxis, cytokinesis, signal transduction, and several aspects of development [3-6]. Additional advantages of *D. discoideum* are easy cultivation allowing large scale cultures and biochemical studies, the amenability to genetic and cell biological analysis and the availability of the genome sequence [2, 7-9]. As a soil amoeba and a phagocyte *D. discoideum* can be a natural host of opportunistic bacteria and may thus have developed strategies to avoid invasion by given pathogens or to counteract their intracellular survival and replication [10-12]. It has already been shown for a number of intracellular bacterial pathogens that they are resistant to free-living amoeba, such as *Acanthamoeba castellanii* [13]. *A. castellanii* occupies the same natural niche as e.g. *L. pneumophila* and *mycobacteria* where selection of virulence traits occurs (see also Sandström *et al*., this issue) [14, 15]. The organism might therefore be considered a closer model than *D. discoideum* to test their virulence, however, *D. discoideum* offers the advantage that mutants can easily be generated [2].

Phagocytosis is a very complex, evolutionarily conserved mechanism that is used by higher eukaryotes to clear dead cells and cell debris and to counter the constant threat posed by pathogens. For this purpose they harbour specialized cells such as macrophages, neutrophils or dendritic cells that have the ability to rapidly and efficiently internalize a variety of organisms and particles and degrade them. These cells represent professional phagocytes that are important for innate and adaptive immunity in metazoa. For lower eukaryotes like *D. discoideum* phagocytosis is a means to internalize bacteria that are used as food source. The ingested microorganism is trapped in a phagosome and, via the phagolysosomal pathway, is ultimately delivered to a lysosome where it is degraded by a cocktail of hydrolytic enzymes [10, 11, 16]. Efficient phagocytosis relies on signalling processes, a functioning cytoskeleton, in particular actin and actin-binding proteins, and vesicle trafficking and fusion. Pathogens, on the other hand, have evolved several means to interfere with these processes. They either block maturation of the phagosome, manipulate its identity and use it as a replication niche or escape from it into the cytosol [17].

In this review we first provide an introduction to *D. discoideum* as a model host for a number of bacterial pathogens followed by a brief description of *L. pneumophila*, *mycobacteria*, and *S. typhimurium*, bacterial pathogens that have been used to study host-pathogen interactions with *D. discoideum*. We then discuss host cell processes that are important for the uptake of the pathogen, the establishment of the replication niche and host defence. We finally address the potential of *D. discoideum* for drug screening.

### *D. discoideum,* a Versatile Model to Study Host Pathogen Interactions

Although Depraitère and Darmon [18] described as early as 1978 that a few bacteria were pathogenic for *D. discoideum*, the system emerged as an experimental model for bacterial infections only ten years ago, when two groups demonstrated that *D. discoideum* could be used as host for *L. pneumophila *[19, 20]. Following these two reports, the number of pathogens for which *D. discoideum* has been shown to be a suitable host has increased steadily, the last entry being *S. typhimurium* (Table **1**). In recent years it became clear that the basic mechanisms of host pathogen interactions are conserved between lower and higher eukaryotes [10, 21, 22]. Moreover, unicellular eukaryotes probably constitute a reservoir in which different pathogenic bacteria survive in the wild and where they develop novel virulence factors that are subsequently effective against animals or humans. Consequently, *D. discoideum* has become an attractive model system for investigating the infection with human pathogens [10-12, 23, 24]. *D. discoideum* cells are very suitable for cell biological assays and imaging, therefore, they have been used to study the dynamics of bacterial uptake, intracellular traffic of the pathogen-containing vacuole and. eventually, bacterial exit. However, the major contribution of *D. discoideum* infection studies resides in the identification of host cell factors that affect infection. To study these factors a large number of *D. discoideum* mutants are available from the *Dictyostelium* stock center (http://dictybase.org/ StockCenter/StockCenter.html), additional genes of interest can be tagged and easily disrupted and also untargeted mutational screens can be carried out [2, 25]. The immense value of the last approach was recently documented by Ralph Isberg’s lab where several new host cell factors that are important for infection with *L. pneumophila* were discovered and analysed [26]. As shown in Table **2**, the list of genes favouring resistance or susceptibility to infection is increasing steadily. In addition, the bacterial side of the coin can easily be studied using *D. discoideum* as a screening host for wild-type or mutagenised pathogenic bacteria followed by a plaque assay (Table **3**) [27, 28]. This approach that will, however, not be discussed in this review, works for pathogenic bacteria that have been already proven to infect *D. discoideum* and allows the fast detection of bacterial virulence genes. For recent reviews see Steinert and Heuner [29] and Weber *et al*. [30].

In the following section we will concentrate on infection studies with *L. pneumophila*, *Mycobacteria* and *S. typhimurium*, whereas for the other pathogens listed in Table 1 the interested reader is referred to a recent excellent review by Margaret Clarke [12]. 

## PATHOGENS THAT INFECT *D. DISCOIDEUM*

### L. pneumophila

In August 1976 a large outbreak of severe pneumonia affected attendees of a convention of war veterans in Philadelphia, USA. The outbreak was caused by a previously unrecognized bacterium and of 182 reported cases 29 were fatal. In early 1977 the causative agent of the “Legionnaires’ disease”, was nailed down and named *L. pneumophila *[31]. The bacterium is Gram-negative and now known as a facultative intracellular parasite*.* Meanwhile, it is clear that *L. pneumophila* is a significant cause of pneumonia. The majority of cases of Legionnaires’ disease are caused by *L. pneumophila* serogroup 1, but other serogroups and other species are also pathogenic [32-35]. *L. pneumophila* infection of alveolar human macrophages usually occurs through inhalation of contaminated aerosols produced by water systems such as air-conditioning units or showers [35]. Upon cell entry the *L. pneumophila* containing vacuole (LCV) is formed but does not enter the endo-lysosomal pathway [36-38]. Instead, a series of alternative docking events take place, including transient recruitment of mitochondria after about 1 hour [39] followed by association of ribosomes after about 4 hours. Then *L. pneumophila* proliferates, becomes acid tolerant and produces a flagellum. After 16 to 20 hours the LCV fuses with lysosomes. Finally, necrosis of the host cell is triggered, which leads to the release of the bacteria [40, 41]. A role of the mitochondria in the infection process is supported by two recent papers with *D. discoideum* as host. In mitochondrially diseased cells *L. pneumophila* could replicate better than in wild-type cells and this was suppressed by inhibiting the expression of the catalytic subunit of the AMP-activated protein kinase (AMPK), the central cellular energy sensor. Conversely, overexpression of the AMPK catalytic subunit enhanced the intracellular growth of *L. pneumophila* [39]. Interestingly, this protein is upregulated in mitochondrial diseases and also upon infection with *L. pneumophila*. By which mechanism AMPK facilitates infection remains unclear. 

Zhang and Kuspa [42] found a decrease of mitochondrial mRNAs already 4h post infection and cleavage of the large subunit of the mitochondrial rRNA into two distinct fragments suggesting that *L. pneumophila* specifically disrupts mitochondrial protein synthesis in *D. discoideum* during the course of infection. Cleavage was particularly pronounced 24 hours post infection and may be correlated with cell death [42].

The pathogenicity of *L. pneumophila* is determined by a number of virulence factors, among them the 24 dot/icm (defect in organelle trafficking/intracellular multiplication) gene products that are responsible for the formation of a type IV secretion system. A large number of effector proteins are transported into the cytoplasm of the host cell and are responsible for the modified phagosome maturation that allows survival of *L. pneumophila* [40, 43]. Genome sequencing of three clinical *L. pneumophila* isolates has revealed new putative virulence factors, among them many eukaryotic-like proteins that are likely to be implicated in different  steps  of  the *L. pneumophila* life cycle [44].  So far no transmission of *L. pneumophila* among humans has been observed and it is assumed that freshwater amoebae and not human alveolar macrophages are the natural host of *L. pneumophila* [40, 45].

To study the infection process of *L. pneumophila*, guinea pigs, different protozoa, monocytes and other human cells have been used, while the suitability of *D. discoideum* was only recognized much later [19, 20, 46]. The infection and replication processes of macrophages and *D. discoideum* with *L. pneumophila* appear very similar. Recent evidence suggests that uptake of *L. pneumophila* into *D. discoideum* occurs by macropinocytosis [47], whereas in macrophages macropinocytosis as well as phagocytosis have been described [48]. However, infection in *D. discoideum* proceeds slower than in macrophages and host cell lysis occurs only after more than 48 hours [49, 50]. Meanwhile a wealth of information about host cell and bacterial factors that are important in the infection process has been obtained with *D. discoideum* as the model system (Tables **2** and **3**, and see below).

### *Mycobacterium tuberculosis* and *Mycobacterium marinum*

There are around 100 different species of *Mycobacteria* which is the only genus in the family of Mycobacteriaceae [51]. *Mycobacteria* have a rod-like appearance and are usually considered Gram-positive. The grouping is based on the lack of an outer cell membrane, though, due to their characteristic cell wall, they do not retain the crystal violet in Gram staining well. Their cell wall is hydrophobic, waxy and thicker than in many other bacteria. It is composed of the hydrophobic mycolate layer and a peptide-glycan layer held together by arabinogalactan. *Mycobacteria* live in water and in the soil, are aerobic, and acid-fast [51]. Several members from the *Mycobacteria* group, including *M. tuberculosis *are human pathogens [35] and cause tuberculosis and other granulomatous lesions: Tuberculosis kills nearly 3 million people annually [52]. Virulence depends among other factors on the region of difference (RD) 1 locus, which encodes components of a type seven secretion system (ESX-1 system) and essential secreted effectors like CFP-10, ESAT-6 [53] and on two large families of proteins, PE and PPE, which could provide antigenic variation to the pathogen in order to evade the host immune response [54-56]. *M. marinum* is a close relative of *M. tuberculosis* and infects amphibians, fishes and also humans [57]. In 1954 *M. marinum* was identified as being responsible for the cutaneous granulomatous lesions of 80 persons who had used the same swimming pool. Therefore, the disease is called swimming pool or fish tank granuloma [58]. *M. tuberculosis* and *M. marinum* share common mechanisms of pathogenicity and the pathologies and lesions they cause are almost indistinguishable [59]. Since *M. tuberculosis* is a biosafety level 3 human pathogen, its study is labor intensive and carries the risk of accidental exposure. Therefore, mycobacterial models like *M. marinum*, *Mycobacterium bovis* (BCG strain) or *Mycobacterium avium* are increasingly used to understand *M. tuberculosis* virulence [60]. On the host side, the mouse is the most commonly used model, however, *M. tuberculosis* is not a natural pathogen of mice and the course of tuberculosis differs from the human disease. In recent years, zebrafish, *D. melanogaster*, *C. elegans* and *D. discoideum* have been firmly established as surrogate hosts [61].

### S. typhimurium 

*Salmonella enterica* serovar Typhimurium is one of more than 2000 species of the *Salmonella enterica* genus, which are resident bacteria of the gut in vertebrates. Only a handful of them are etiological agents of gastroenteritis and the more severe typhoid fever. Typhoid fever, which is characterized by fever, intestinal perforation and hemorrhage, enlargement of mesenteric lymph nodes, spleen and liver, is caused mostly by *S. enterica* serovar Typhi, which is a human pathogen that does not cause disease in other animals.

*S. typhimurium* is spread in both animals and humans and is the major agent of food-borne (mainly meat and eggs) gastroenteritis, a disease characterized by diarrhea, abdominal pain, nausea, vomiting and fever. Acute enteritis may last for up to a week and resolves spontaneously, but the disease is a major economic problem. In contrast to *S. typhi* which is endemic in Asia, Africa and South America, *S. typhimurium* is widespread also in Europe and North America, with an estimate of 1.4 million cases of enterocolitis, including 550 annual deaths, in the USA alone [62]. 

Established animal model systems for *S. typhimurium* are the mouse and *C. elegans*. In the mouse, for which *S. typhimurium* is a natural pathogen, the symptoms resemble those of typhoid fever in humans, which include enlargement of mesenteric lymph nodes, spleen and liver and eventually sepsis. After colonization of the intestinal epithelium, the bacteria are internalized by resident macrophages in the submucosa and rapidly disseminate by infecting circulating macrophages, B and T cells and eventually colonizing resident phagocytes in liver and spleen [62].

*S. typhimurium* internalization occurs by phagocytosis or macropinocytosis. Phagocytosis is common in professional phagocytes, and is induced by binding to lipopolysaccharide, fimbriae or flagellin receptors. Macropinocytosis is, instead, a highly specific bacterium-induced process for entering non-professional phagocytes as well as phagocytes. The process is regulated by the type 3 secretion system (T3SS), a protein complex encoded in the SPI1 (Salmonella pathogenicity island 1) gene locus, that secretes several effectors in the cell, inducing re-organization of the actin cytoskeleton, with formation of massive localized membrane ruffles and macropinocytic cups [63-67]. The outcome of infection depends on the modality of uptake, with macropinocytosis leading preferentially to formation of a survival and replication niche, the Salmonella-containing vacuole (SCV), whereas bacteria taken up by phagocytosis are mostly transported to lysosomes. The SCV is initially characterized by acquisition of early endosomal markers, which are removed and substituted within 60 to 90 minutes by late endosomal and lysosomal markers [68, 69]. Maturation of the SCV and virulence are controlled by the SPI2 T3SS system, a second secretion system that secretes hundreds of proteins into the cytoplasm [67, 70-73]. Boucrot *et al*. showed that the SCV recruited the plus-end-directed motor kinesin and that this event was regulated by proteins translocated by the SPI2 T3SS, among them SifA [74]. Interestingly the early SCV migrated to the perinuclear area and escaped the fusion with lysosomes [75].

*S. thyphimurium* is phagocytosed by *D. discoideum* amoebae almost as well as *E. coli* B/r. An earlier report suggested that the bacterium was not pathogenic for *D. discoideum* [76]. Jia *et al*. [77] reported that *D. discoideum* knockout mutants for autophagy genes atg1, atg6 or atg7, in contrast to control cells, supported establishment of a replicative niche, suggesting that autophagy was required for *S. typhimurium* degradation. By using a DNA microarray approach, a different pattern of RNA expression was found, in comparison to non-pathogenic bacteria, suggestive of cells entering starvation, despite the fact that *S. typhimurium* was ingested. The starvation response of the cells and its potential subversion by *S. typhimurium* is under study (Sillo *et al*., unpublished results).

## CRUCIAL HOST CELL PROCESSES

### Phagocytosis and Macropinocytosis 

Invasive bacteria exploit phagocytosis or macropinocytosis to enter the cells. Both processes are characterized by the formation of relatively large vesicles on the plasma membrane, which are regulated by localized recruitment of the actin cytoskeleton. Phagocytosis is induced by membrane signalling triggered by particle binding to specialized membrane receptors and leading to tight enveloping of the particle by the protruding plasma membrane. Macropinocytosis is usually a cell autonomous process, resulting in massive recruitment of actin beneath the membrane, formation of ruffles and vesicles of variable size filled with extracellular liquid. Bacteria or other particles present in the external milieu can be engulfed with the liquid independently of any specific binding [78-80]. Macropinocytosis can also be induced in non-professional phagocytes by some pathogens to enter the cell. The process has been described for *Salmonella*, *Mycobacteria* and *Legionella* [47, 48, 78, 81, 82].

In macrophages, receptors involved in phagocytosis include the Fc receptor family, the complement receptor (CR3) and lectins [80, 83, 84]. The best known case in macrophages is the signalling pathway linked to the Fcγ receptor. Particle binding leads to receptor clustering and phosphorylation by Src-family kinases, generating docking sites for the Syk kinase, which in turn facilitates binding of docking proteins and PI3K, leading to actin cytoskeleton reorganization [84, 85]. In *D. discoideum,* the heterotrimeric Gα4βγ protein mediates membrane signals leading to phagocytosis, possibly resulting from receptor clustering. The *D. discoideum* bona fide phagocytosis receptors are so far unknown [10, 86, 87]. However, adhesion molecules like Phg1, SibA and SadA have been described and it is likely that one or a few of them are adhesion molecules involved in phagocytosis [88-90].

A major role in actin re-organization in the phagocytic and macropinocytic cup is played by membrane phosphoinositides, particularly PI(4,5)P_2_. This phosphoinositide is the most abundant PI-form of the plasma membrane and recruits several PH-domain containing proteins, among which are the regulators of actin nucleation, such as the Arp2/3 complex, WASP and WAVE, small G proteins of the Rho family and actin binding proteins [91]. Disappearance of PI(4,5)P_2_ is due to the activity of enzymes such as PI-PLC, PI3K or the PI-5-phosphatase, and is a pre-requisite for actin coat disassembly, vesicle closure and further fusion with vesicles of the endo-lysosomal pathway [92-95]. Both in macrophages and *D. discoideum*, PI-PLC inhibitors completely inhibit phagocytosis of bacteria, such as *E. coli*, as well as macropinocytosis, whereas PI3K inactivation interferes with phagocytosis of larger particles or with macropinocytosis [47, 86, 92, 94-98]. Actin assembly during phagocytosis is also regulated by small G proteins of the Rac subfamily, which activate WASP/WAVE family proteins [99, 100]. In *D. discoideum* there are 18 genes encoding Rac proteins, some of which are involved in phagocytosis or macropinocytosis. Except for RacH, however, which appears to regulate macropinocytosis, but not phagocytosis [101], the results obtained with null mutants and overexpressors for other *rac* genes underline a high degree of redundancy that explains the absence of phenotypes when a single gene is disrupted [10]. In *D. discoideum*, macropinocytosis is responsible for the vast majority of pinocytic events [79], and is gain of function due to a few nitrosoguanidine-induced mutations in some axenic strains [1]. The differential requirement for macropinocytosis between wild type natural isolates and axenic strains has recently allowed us to show that *L. pneumophila*, in contrast to other pathogens, such as *M. avium* or *M. marinum*, *Neisseria meningitides* or *S. typhimurium*, is taken up exclusively by macropinocytosis [47].

### Phagosome Maturation

In less than 5 minutes after engulfment of non-pathogenic bacteria, yeast particles or latex beads, the phagosome or macropinosome fuses with acidic vesicles harbouring the V-H^+^ ATPase and with vesicles decorated with the Nramp1 protein [102-105]. Fusion with acidic vesicles appears to be regulated, both in *D. discoideum* and macrophages, by PI(3)P, a PI-form generated mainly via class III PI3K [106-108]. PI3K modulates recruitment of the small G proteins Rab5 and Rab7 to phagosomes, and PI3K inhibitors block phago-lysosome biogenesis [109]. In macrophages, Rab5 is rapidly recruited to newly formed phagosomes and is necessary for the subsequent enrollment of Rab7 either from a soluble pool or by fusion with Rab7-containing endosomes. Acquisition of Rab7 favours recruitment of motor proteins, transport of phagosomes toward the MTOC and fusion with late endosomes and lysosomes [109-111]. Rab7 regulates phagosome fusion with lysosomes, but not with acidic vesicles, not only in macrophages but also in *D. discoideum* [102, 112], where RabD (homolog of Rab14) appears to stimulate vesicle homotypic fusion, leading to formation of large vesicles containing several bacteria [113]. Studies using invasive and non-invasive *Salmonella enterica *serovar Typhimurium have shown that several other Rab proteins, in addition to Rab5, 7 or 14, associate selectively with wild type or mutant *S. typhimurium*, some of which are necessary for phagosome maturation [114]. It appears that phago-lysosome biogenesis is a process involving several small Rab GTPases and cannot be explained only by the single transition between Rab5 and Rab7.

The participation of small GTPases in phagosome maturation is also supported by a recent proteome analysis of *L. pneumophila* vacuoles purified by magnetic immunoseparation and density gradient centrifugation. Mass spectrometric analysis of purified LCVs revealed 566 host cell proteins, among them known LCV components such as the small GTPases Arf1, Rab1 and Rab7 and novel components such as Rab8, an endosomal regulator of the late secretory pathway, and the endosomal GTPase Rab14. The authors conclude that LCVs also communicate with the late secretory and endosomal pathways [115]. In a parallel study Shevchuk *et al*. identified in classically purified LCVs 157 host proteins which belong to different functional categories among them a number of cytoskeletal proteins, subunits of the vacuolar ATPase, proteins involved in the stress response and of the proteasome system but no small GTPases, as described above [116].

In order to survive and to establish a replicative niche, pathogens must interfere with the maturation process. They do so by either i) slowing down or stalling maturation, ii) changing the route of the phagosome or iii) escaping from it into the cytosol [17]. Another survival strategy is adaptation to the bactericidal, acidic lysosomal compartment, which is the case for *Coxiella burnetii,* the agent of Q fever [117, 118].

**Fig. (1). Infection of D. discoideum with different pathogens.**
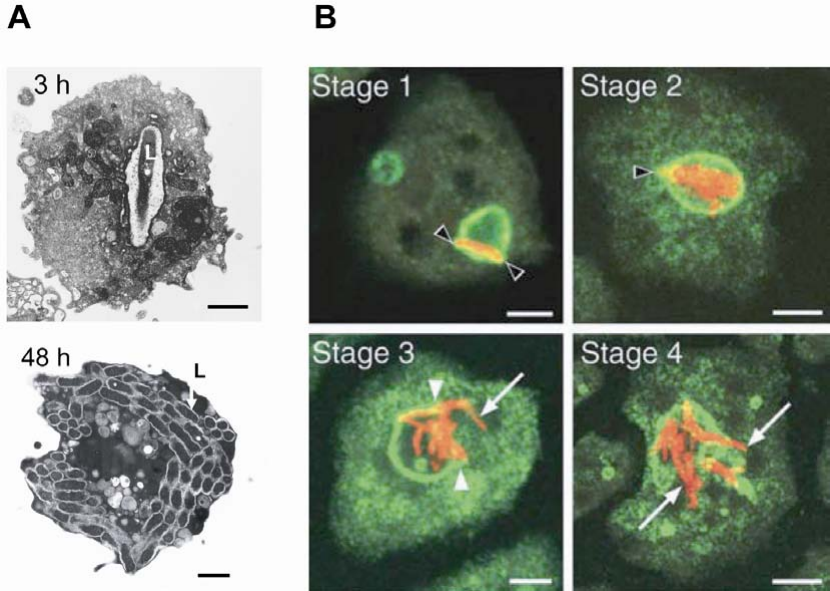
**A)** Transmission electron micrographs of *L. pneumophila* PhilI JR32 infected *D. discoideum* cells 3 and 48 hours post infection. 3 h after infection the host cell contains mostly one *L. pneumophila* (L) within the phagosome. After 48 h the *D. discoideum* cell is almost entirely filled with *L. pneumophila*. Scale bars, 2 µm. (*Reproduced from figure 1 of [149], modified*). **B)** Immunofluorescence micrographs of phase 2 of *M. marinum* infection of *D. discoideum*. Four sequential stages can be distinguished in the establishment and rupture of the vacuolin-positive vacuole. At the early stage 1, a single mycobacterium deformed a vacuole already enriched in vacuolin (black arrowheads). The second stage is defined by the proliferation of the bacteria inside the vacuole which leads to more deformation of the membrane (black arrowhead). At the late stages 3 and 4, the vacuolin-positive membrane was ruptured (arrowheads mark the edges of the membrane sheets generated during niche rupture) and bacteria were released into the cytosol (arrows). *M. marinum* is labelled in red and vacuolin in green. The scale bar represents 5 µm (*Reproduced from figure 3 of [121], modified*).

We will first consider results with *M. tuberculosis* and *M. marinum. *After uptake by macrophages or by *D. discoideum,* the pathogen prevents the maturation of the phagosome and replicates inside a compartment that resembles an early endosome [119]. The arrested mycobacterium containing vacuole (MCV) is characterized by the presence of early endosomal markers, the lack of late endosomal or lysosomal markers like the V-ATPase and diminished PI(3)P levels (for review see [120]). Hagedorn and Soldati [121] divided the proliferation of *M. marinum* in *D. discoideum* in three distinct phases i) an initial lag phase until 12 hpi, ii) a major proliferation phase from 12 -37 hpi and iii) a plateau or decrease in the cfu after 37 hpi. They further could divide the proliferation phase into four stages (Fig. **1**). In the early stage 1, a single mycobacterium resides in a vacuole enriched in vacuolin. The second stage is defined by the proliferation of the bacteria. At the late stages 3 and 4, the vacuolin-positive membrane is ruptured and bacteria are released into the cytosol [121]. After that, *M. marinum* and *M. tuberculosis,* but not *M. avium,* can spread to neighbouring cells via a non-lytic mechanism that requires the host cytoskeleton and an intact mycobacterial ESX-1 secretion system [122]*.*

In contrast, *L. pneumophila* changes the route of the *Legionella* containing vacuole (LCV) and is found in a compartment that is different from that of a non-pathogen. The LCV first associates with mitochondria and with vesicles derived from the ER. It then binds ribosomes and becomes similar to rough ER (for review see [24]). The LCV is characterised by the ER resident protein calnexin, the v-SNARE Sec22b and the small GTPases Arf1 and Rab1 [123, 124]. Finally the calnexin-positive LCVs undergo a transition from tight to spacious vacuoles a few hours post-infection [50]. In *D. discoideum* the LCV recruits quite rapidly Nramp1, but not the V-H^+^ ATPase nor vacuolin. Only late in infection are the V-H^+ ^ATPase or the post-lysosomal marker vacuolin found in large vacuoles containing replicating bacteria [47]. Whether *L. pneumophila* uses the post-lysosomal pathway for exiting the cell, as shown for *mycobacteria*, is unclear; extensive cell lysis occurs 48 hours post-infection, which suggesta that the bacteria leave the cells by lysing them.

It turned out that the PI metabolism is critically involved in these processes as *L. pneumophila* secretes effector proteins via the Icm/Dot type 4 secretion system that bind to PI(4)P on the LCV [125-127]. Furthermore, bacterial replication was more efficient in *D. discoideum* cells lacking the inositol polyphosphate 5-phosphatase, Dd5P4, a homologue of human OCRL1 (Oculocerebrorenal syndrome of Lowe), implicated in retrograde endosome to Golgi trafficking [30], and in *D. discoideum* mutants of phosphatidylinositol-3 kinases (PI3Ks) and PTEN [47, 125]. Interestingly, inactivating PI3K has no effect on calnexin or Nramp1 recruitment to LCV, whereas fusion with acidic vesicles is further blocked, suggesting that *L. pneumophila* may hinder V-H^+ ^ATPase recruitment by altering the phosphoinositide composition of the LCV, thus favouring formation of a replication vacuole [47]. 

In *D. discoideum *Rab14 induces phagosome homotypic fusion, leading to formation of large vesicles. In macrophages, Rab14 silencing or expression of Rab14 dominant-negative mutants lead to phagolysosomal maturation of phagosomes containing live mycobacteria, whereas overexpression of Rab14 or of a constitutively active Rab14 mutant blocks maturation of phagosomes containing dead bacteria [128]. Similarly, Rab22 that is transiently expressed on latex beads containing phagosomes was, instead, retained on *M. tuberculosis*-containing phagosomes [129]. Therefore the presence of Rab14 or Rab22 in macrophages seems to be important to inhibit or delay phago-lysosomal biogenesis.

Ion acquisition is important for intracellular survival of pathogenic bacteria. Mg^2+^, Mn^2+^, K^+^ and Zn^2+^ have been implicated in *S. typhimurium* virulence [130-133], whereas Fe^2+ ^is an essential metal for all cells, and it is known that *Salmonella*, *Legionella* and *Mycobacteria* accumulate large amounts of iron [134-136]. In response to iron deprivation, these bacteria express siderophores to recruit iron. Iron availability in the phagosome is limited by the activity of Nramp1, a divalent metal transporter that depletes the phagosome of iron by a mechanism dependent on the proton gradient [105, 137]. Mutations in Nramp1 have been linked to innate susceptibility to mycobacterial diseases and *S. typhimurium* infection [137-140]. Inactivation of the gene in *D. discoideum* leads to increased intracellular growth of *L. pneumophila* and *M. avium*, whereas its overexpression completely inhibits *L. pneumophila* growth [105]. *L. pneumophila* hinders recruitment of the V-H^+^ ATPase in the *Legionella*-containing vacuole, without interfering with Nramp1 recruitment. Since a proton gradient is required for Nramp1-dependent depletion of iron, the absence of the vacuolar ATPase generates a milieu in which Nramp1 does not function properly [47]. 

### Macroautophagy

Bacterial pathogens manipulate host cell processes to avoid phago-lysosomal fusion and to establish a replicative niche [24]. The host, on the other hand, initiates elaborate defense processes, of which one appears to be macroautophagy (hereafter autophagy) [141]. Autophagy is an ancient cellular pathway that is conserved from yeast to humans and has presumably evolved to enable cells to survive periods of starvation. More than 30 autophagy (ATG) genes have been identified, mainly in yeast, of which 18 constitute the core machinery for starvation induced autophagy. Cytosolic material is captured into double membrane-bound vesicles that mature into autophagosomes and then, after fusion with lysosomes, become autophagolysosomes. There, the cargo is degraded and then recycled for further use [142]. Autophagy contributes to many physiological and pathological processes, including cell differentiation and development, programmed cell death, cancer and neurodegenerative disorders. There is accumulating evidence that autophagy is also a general and important defense mechanism in the complex interactions between host and pathogen [143]. Some pathogens e.g. *M. tuberculosis* are targeted for degradation through autophagy [144]. In a recent genome-wide analysis of the host intracellular network that regulates survival of *M. tuberculosis *it was found that host factors predominantly function through the regulation of autophagy [145]. Other pathogens have developed means to evade autophagy, e.g. *Shigella flexneri* or even to utilize the autophagosome for replication e.g. *Staphylococccus aureus* [141, 146, 147]. 

In *D. discoideum* the role of autophagy in infection was so far investigated with *L. pneumophila* and *S. typhimurium* in several autophagy mutants. Otto *et al*. reported for atg1, 5, 6, 7 and 8 knock-out mutants that autophagy is dispensable for intracellular *L. pneumophila* replication. However, the authors did not examine if autophagy might be important in restricting the intracellular replication [148]. A microarray study of the time course of *Legionella* infection revealed differential regulation of three core autophagy genes that are required in the early phase of autophagosome formation [149]. Interestingly, ATG9 was up-regulated while ATG8 and 16 were down-regulated, suggesting that host and pathogen target different pivotal autophagy genes during infection. It is tempting to speculate that the host tries to up-regulate autophagy via ATG9 while the pathogen counteracts via down-regulation of ATG8 and 16. A knock-out mutant of the ATG9 gene showed a strong phagocytosis defect that was particularly apparent when cells were infected with *L. pneumophila*. However, those Legionellae that entered the host could multiply better in mutant than in wild-type cells. This was due to a less efficient clearance in the early phase and a more efficient replication in the late phase of infection [150]. In an elegant recent study two model organisms, *Caenorhabditis elegans* and *D. discoideum*, were used to examine the effects of autophagy gene inactivation on infection with *S. typhimurium*. In both organisms, the inactivation of autophagy genes increased the intracellular replication of *S. typhimurium* [77]. In support of a role of autophagy in infection with *L. pneumophila *it is also worth mentioning that the LCV is associated with markers of autophagy, such as ATG7 and 8. If the LCV is a modified autophagosome, then autophagy must be arrested for the bacteria to maintain intracellular replication [151]. Thus, consistent with studies using macrophages and other models the data from *D. discoideum* support a protective role of autophagy during pathogen infection, raising the possibility that cellular defense against pathogens could be induced by drugs that stimulate autophagy.

## *D. DISCOIDEUM* AS EXPERIMENTAL SYSTEM FOR DRUG TESTING

As mentioned in the previous sections, *D. discoideum* cells share with higher eukaryotes several cellular processes and underlying homologous genes [9]. In addition, the cells are not encased in a rigid cell wall and the plasma membrane is thus directly exposed to the extracellular milieu. The composition of the plasma membrane is not basically different from that of higher eukaryotes, except that cholesterol is substituted with ergosterol and that, among the carbohydrate residues of proteins or glycolipids, sialic acid is not found [152]. It is therefore not surprising that pharmacological approaches have been regularly used with *D. discoideum* cells and that many drugs affecting mammalian cells have proven to be effective also in *D. discoideum*, though in some cases higher concentrations are required. 

PLC and PI3K inhibitors, such as U73122, wortmannin, or LY294002, have been used to characterize phagocytosis and macropinocytosis [86, 97] as well as chemotaxis [153-155]. Actin assembly can be inhibited by cytochalasin or latrunculin A, thus inhibiting spontaneous and chemotactic cell motility as well as phagocytosis and macropinocytosis [156-159]. PLA2 inhibitors do not affect phagocytosis [86, 97], but they have been shown to inhibit calcium signalling [160] and, when used in combination with PI3K inhibitors, chemotaxis [155, 161]. Intracellular and extracellular calcium chelators, such as BAPTA-AM or EDTA and EGTA, have been used to study, among others, cell-cell adhesion and phagocytosis [86, 162-164]. Tyrosine kinase and phosphatase inhibitors helped in showing actin phosphorylation changes [165-168]. Valproic acid or cisplatin have been used to study lithium signalling and effects on gene expression, growth and development [169-172]. 

In infection studies, drugs have been used to characterize *L. pneumophila* uptake and replication [173]. Pharmacological or genetic inhibition of PI3K stimulates *L. pneumophila* infection [47, 125]. Addition of the PI3K inhibitor LY294002 at different time points during infection has recently been used to identify a short period immediately after bacterial uptake, which is sensitive to addition of the drug, stimulating intracellular replication of the bacteria [47]. Similarly, it has been shown pharmacologically that PLC and actin assembly are required for *L. pneumophila* uptake, but do not seem to play a role for establishment of the replicative niche [47]. 

These scattered and largely incomplete examples emphasize that *D. discoideum* cells can be conveniently used for drug testing [174, 175], in combination with the variety of assays that have been developed to study phagocytosis, infection as well as cell motility, chemotaxis, cell-substratum and cell-cell adhesion, signalling, growth, cell differentiation or development [164]. 

## CONCLUSIONS

Investigations with model organisms have significantly contributed to our understanding of host-pathogen interactions and have lead to the discovery of many host genes that are either involved in the defence response or required for the pathogen to establish its replicative niche. *D. discoideum* is particularly suited for infection studies, because it is a professional phagocyte, its genome is completely sequenced and excellent genetic, biochemical and cell biological tools are available  [2, 8, 10, 11]. Mainly, *D. discoideum* was used to study the host response upon infection with different pathogens in particular *L. pneumophila*, *M. marinum* and *M. avium* and *S. typhimurium. *This led to the discovery of a variety of bacterial and host cell factors, among them many genes encoding cytoskeletal and signaling proteins that are important in the infection process. A further advantage of *D. discoideum* is that it can be easily used for drug testing and as screening host for wild-type or mutagenised pathogenic and non-pathogenic bacteria [27, 28]. Furthermore, untargeted mutational screens to find crucial host factors can be carried out [26]. In summary, the properties of *D. discoideum* in combination with the impressive armoury of tools that is available will help to further dissect host pathogen crosstalk in the years to come.

## Figures and Tables

**Table 1:** Bacteria that have been Successfully Used to Infect *D. discoideum*

Bacterial Pathogen	References
*Legionella pneumophila*	[20, 49]
*Mycobacterium avium, M. marinum, M. tuberculosis*	[76, 121, 176]
*Pseudomonas aeruginosa*	[27, 76, 177]
*Vibrio cholerae*	[178]
*Klebsiella pneumoniae*	[179]
*Neisseria meningitidis*	[180]
*Burkholderia cenocepacia*	[181]
*Salmonella typhimurium*	[77]

**Table 2:** Host Cell Factors that Affect *D. discoideum*-Pathogen Interactions

Host Cell Factor	Approach	Effects on Infection		Pathogen	References
		Uptake	Growth		
F Actin	inhibitors	down	normal	L.p.	[47]
α-actinin/ABP120	knockout	down	down	L.p.	[173]
Coronin A	knockout	down	normal	L.p.	[173]
		up	up*	M.m.	[176]
Coronin B	Knockout	up	normal	L.p.	[182]
	overexpression	down	normal		
Myosin1(A/B)	knockout	normal	up	L.p	[49]
Profilin I/II	knockout	normal	up	L.p.	[20]
Daip1	knockout	down	normal	L.p.	[173]
Villidin	knockout	down	down	L.p	[173]
Lim C/D	knockout	down	down	L.p	[173]
Comitin	knockout	down	up	L.p.	[183]
Calnexin	knockout	down	down	L.p	[173]
Calreticulin	knockout	down	down	L.p.	[173]
Gβ subunit	knockout	down	down	L.p.	[173]
RacH	knockout	down	up	M.m.,	[121]
		down	up	L.p.	[47]
PLC	inhibitors	down	normal	L.p.	[47]
Calcium level	inhibitors	down	n.t.	L.p.	[173]
PI3K1/2	knockout	normal	up	L.p.	[125]
PI3K1-5	knockout	down	up	L.p.	[47]
PI3K1-5/PTEN	knockout	down	up	L.p.	[47]
PTEN	knockout	down	down	L.p.	[47]
Dd5P4 (OCRL1)	knockout	down	up	L.p	[30]
Phg1	knockout	normal	up	K.p.	[179]
Nramp1	knockout	normal	up	L.p., M.a	[105]
	overexpression	normal	down		
VacB (flotillin)	knockout	normal	down	L.p.,M.m.	[121]
RtoA	knockout	normal	down	L.p.	[184]
Kil1	knockout	normal	up	K.p.	[179]
	overexpression	normal	down		
TirA	knockout	n.t.	up	L.p.	[185]
Rnl, hsp60	KO/antisense	normal	up	L.p.	[39]
AMPK	overexpression	normal	up	L.p.	[39]
	antisense	normal	normal		
ATG1, 6, 7	knockout	normal	up	S.t.	[77]
ATG9	knockout	down	up	L.p.	[150]
DupA	knockout	n.t.	down	L.p., M.m	[26]

Pathogen uptake and intracellular growth in *D. discoideum* mutants or upon treatment of wild type cells with inhibitors (F actin: cytochalasin A, latrunculin A; phospholipase C: U73122; intracellular calcium levels: BAPTA-AM, Thapsigargin). Effects ("up" or "down") on bacterial uptake or intracellular growth are relative to AX2 control cells, in the case of inhibitors, or to the parental strain, in the case of mutants. n.t.: not tested.

* Solomon *et al*. 2003 reported an enhanced initial rate of replication until day 4 in comparison to the AX2 control cells. L.p., *L. pneumophila*; K.p., *K. pneumoniae*; M.a, *M. avium*; M.m., *M. marinum*; S.t., *S. typhimurium*.

**Table 3:** *D. discoideum* as Screening Host for Microbial Genes

Gene	Pathogen	References
*dotH, dotI, dotO*	*L. pneumophila*	[19]
*lepA, lepB*	*L. pneumophila*	[186, 187]
*sdhA*	*L. pneumophila*	[188]
*vipD*	*L. pneumophila*	[189]
*lqs, rpoS, letA*	*L. pneumophila*	[190-192]
*sidJ*	*L. pneumophila*	[193]
*enhC*	*L. pneumophila*	[194]
*sidC, sdcA, sidM*	*L. pneumophila*	[126]
*legC3*	*L. pneumophila*	[195]
*rpoS*	*L. pneumophila*	[196]
*ankB*	*L. pneumophila*	[197]
*lpnE*	*L.pneumophila*	[30]
*vas*	*V. cholerae*	[178]
*lasR, rhl, pscJ, exoU*	*P. aeruginosa*	[27, 177]
*trpD, pchH, pchI*	*P. aeruginosa*	[198]
*Rd1*	*M. marinum*	[122]
